# Pycnodysostosis with novel gene mutation and sporadic medullary thyroid carcinoma: A case report

**DOI:** 10.1097/MD.0000000000008730

**Published:** 2017-12-15

**Authors:** Xiulin Shi, Caoxin Huang, Fangsen Xiao, Wei Liu, Jinyang Zeng, Xuejun Li

**Affiliations:** a Department of Endocrinology and Diabetes; b Xiamen Diabetes Institute, The First Affiliated Hospital of Xiamen University, Xiamen, Fujian, China.

**Keywords:** cathepsin K, medullary thyroid carcinoma, pycnodysostosis

## Abstract

**Rationale:**

Pycnodysostosis is a rare autosomal recessive skeletal dysplasia caused by a mutation in the cathepsin K encoded by cathepsin K gene (*CTSK*). Medullary thyroid carcinoma (MTC) is also a relatively rare type of primary thyroid carcinoma.

**Patient concerns:**

A 31-year-old woman presenting a short stature and a palpable nodule in the front of her neck that had gradually increased in size during the last 2 years was referred to our department. She has experienced multiple fractures at lower limbs in the last 2 decades.

**Diagnoses:**

The patient's clinical examination revealed short stature, underweight, a prominent forehead, stubby fingers, and a fixed nodule in the right thyroid lobe. Intraoral examination revealed multiple clinically malposed and missing teeth, as well as chronic periodontitis with a narrow and grooved palate. Radiographic examination revealed typical widely separated cranial sutures and an open anterior/posterior fontanel with an obtuse gonial angle, acroosteolysis, and osteosclerosis with narrowed medullary cavities. Ultrasonography of the thyroid gland showed a marked hypoechoic solid nodule in the right lobe in which tumor cell clusters were confirmed by ultrasound-guided fine needle aspiration biopsy and was suspected to be MTC. Laboratory tests revealed dramatically elevated serum calcitonin >2000 pg/L (reference range: 0–5 pg/L) and carcinoembryonic antigen (CEA) 134.37 ng/mL (reference range: 0–5 ng/mL). Genotypic screening revealed compound heterozygous mutations in the *CTSK* gene (c.158delA, P.Asn53Thr/c.C830T, P.Ala277Val) but no mutation associated with the familial forms of MTC.

**Interventions:**

The patient underwent a total thyroidectomy with right-sided functional neck dissection.

**Outcomes:**

CEA and serum calcitonin decreased significantly postthyroidectomy, and no further fracture has been reported by the patient so far.

**Lessons:**

The present study is the first to report a rare case of the coexistence of pycnodysostosis with a compound *CTSK* gene mutation and sporadic MTC. Radiological techniques and gene analysis play key roles in the definitive diagnosis.

## Abstract 2

Supplemental Digital Content is available in the text

## 1 Introduction

Pycnodysostosis (OMIM 265800) is a rare inherited osteosclerotic skeletal disease first described and named by Lamy and Maroteaux in 1962.^[1]^ To date, only approximately 200 cases have been reported (an estimated prevalence of 1–1.7 per million) with the typical features including short stature; a typical dysmorphic appearance with a particular cranial conformation; cranial dysplasia; short and stubby fingers; an obtuse angle of the mandible; and increased bone density, osteosclerosis, bone fragility with frequent fractures, as well as dental abnormalities. This disorder is caused by a homozygous or compound heterozygous mutation in the cathepsin K gene (*CTSK*), which encodes a lysosomal cysteine protease highly expressed in osteoclasts. Medullary thyroid carcinoma (MTC) is also a relatively rare type of primary thyroid carcinoma originating from the parafollicular C cells. MTC accounts for only 5% to 10% of all thyroid carcinomas^[2]^; however, it exhibits a more aggressive behavior, which increases the difficulty of treatment. MTC can be either sporadic or familial, which is defined as part of the cancer syndrome known as multiple endocrine neoplasia type 2 (MEN2). To our knowledge, no case of pycnodysostosis coexisting with MTC has been reported. Herein, we describe a case of coexistence of these 2 rare diseases.

## 2 Case report

**Figure 1:**
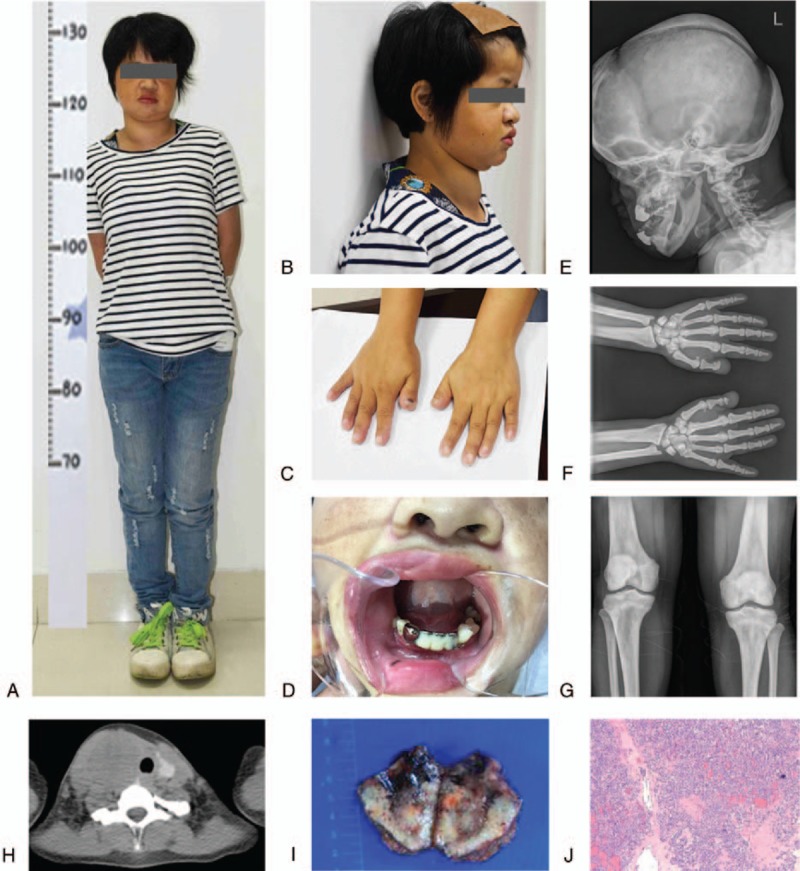
A routine clinical examination revealed the patient's short stature at 132 cm, with the upper segment at 70 cm and the lower segment at 62 cm (A); prominent forehead (B); stubby fingers (C) and a fixed nodule (6 cm in diameter) in her right thyroid lobe (B); intraoral examination revealed multiple clinically malposed and missing teeth and chronic periodontitis with a narrow and grooved palate (D); radiographic examination revealed paranasal sinuses that were nonpneumatized with an obtuse angle of the mandible (E); acroosteolysis (F); and osteosclerosis (G) with narrowed medullary cavities (H). Postoperative histopathology confirmed medullary thyroid carcinoma (MTC) in the right lobe of the thyroid, with extrathyroidal extension and right-sided neck metastases in which 7 out of 9 nodes were positive for tumor (I–J).

**Figure 2:**
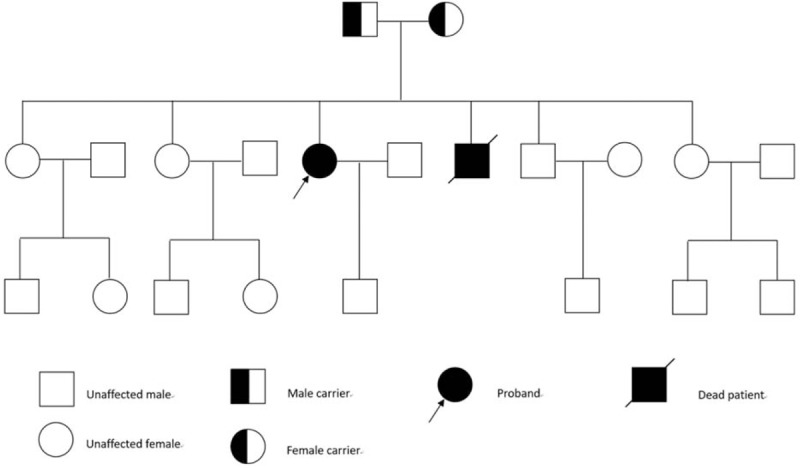
The pedigree of the patient's family. The proband was the 3rd of 6 siblings in a nonconsanguineous Chinese family. Her parents, 2 elder sisters, 1 younger sister, and 1 younger brother had a normal height and weight; however, the other younger brother displayed a similar short stature and died in an accident at the age of 14. Other members of this family were neither affected nor displayed clinical manifestations.

A 31-year-old woman was referred to our department with a history of short stature and a palpable nodule in the front of her neck that had gradually increased in size during the last 2 years. She experienced minor falls twice at the age of 24 and 28 and was diagnosed with multiple fractures of the lower limbs, which were treated conservatively. Upon admission, a routine clinical examination revealed that the patient's standing height was 132 cm with the upper segment at 70 cm and the lower segment at 62 cm (Fig. 1A), and she was also underweight (body weight: 33.2 kg). Clinical examination also revealed a prominent forehead (Fig. 1B), stubby fingers (Fig. 1C), and a fixed nodule (6 cm in diameter) in her right thyroid lobe (Fig. 1B). Intraoral examination revealed multiple clinically malposed and missing teeth, as well as chronic periodontitis with a narrow and grooved palate (Fig. 1D). Moreover, retracted bilaterally temporomandibular joints and scoliosis were noted. There was no significant pallor or hepatomegaly. The pedigree of her family is shown in Fig. 2. The proband (the patient) was the 3rd of 6 siblings in a nonconsanguineous Chinese family. She was born after a full-term pregnancy and normal delivery, but her length and weight were lower than others of the same age and sex. One of her younger brothers displayed a similar short stature; however, he died accidentally at the age of 14 after being crushed while playing with other children. All the other members of this family, including her parents, were not affected.

Laboratory tests revealed that the complete blood count, electrolytes, renal and liver function, urinalysis, serum thyroid-stimulating hormone (TSH), free triiodothyronine, free thyroxine, growth hormone, insulin-like growth factor 1, and cortisol were normal. Notably, dramatically elevated serum calcitonin >2000 pg/L (reference range: 0–5 pg/L) and carcinoembryonic antigen (CEA) 134.37 ng/mL (reference range: 0–5 ng/mL) were detected. Radiographic examination revealed widely separated cranial sutures and open anterior and posterior fontanels. The paranasal sinuses were nonpneumatized with an obtuse angle of the mandible (Fig. 1E). Acroosteolysis (Fig. 1F) and osteosclerosis (Fig. 1G) with narrowed medullary cavities (Fig. 1H) were also observed. Ultrasonography of the thyroid gland showed a marked hypoechoic solid nodule (6.2 cm in diameter) in the right lobe. Ultrasound-guided fine needle aspiration biopsy of this thyroid nodule revealed tumor cell clusters suspected to be MTC. Consequently, the patient underwent a total thyroidectomy with right-sided functional neck dissection. Postoperative histopathology confirmed MTC in the right lobe of the thyroid with extrathyroidal extension and right-sided neck metastases in which 7 out of 9 nodes were positive for tumor (Fig. 1I, J).

**Figure 3:**
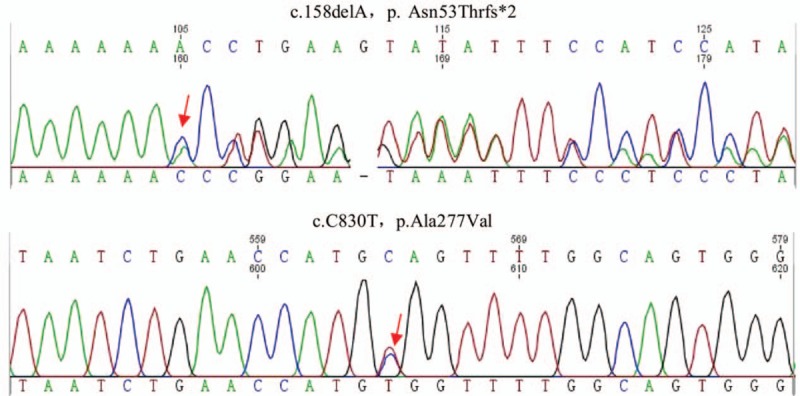
Sequencing diagrams of the compound heterozygous mutations. In this study, 2 heterozygous variants of *CTSK* were detected by Sanger sequencing. One was a novel frameshift variant in exon 3 (c.158delA [P.Asn53Thrfs∗2]) and the other was a missense variant in exon 7 (c.C830T [P.Ala277Val]). Ala = alanine, Asn = asparagine, *CTSK* = cathepsin K gene, Thr = threonine, Val = valine.

Genotypic screening of the whole blood revealed compound heterozygous mutations in the *CTSK* gene (c.158delA, P.Asn53Thr/c.C830T, P.Ala277Val [Asn, asparagine; Thr, threonine; Ala, alanine; Val, valine]) (Fig. 3), but no mutation in the rearranged during transfection gene (*RET*) proto-oncogene associated with familial forms of MTC. A further evaluation was performed postthyroidectomy, revealing that CEA and calcitonin deceased significantly – serum calcitonin at 862 pg/L and CEA at 6.33 ng/mL. One year after total thyroidectomy, laboratory test showed calcitonin at 1149 pg/L and CEA at 8.52 ng/mL, as well as swollen lymph nodes in the neck revealed by ultrasonography. Thus, lymph node metastases consequent to MTC was considered. This patient has been referred to the Department of Oncology for further treatment. Key milestones of the diagnoses and interventions were depicted in timeline figure (supplemental digital content).

Based on the characteristic clinical, radiological, and histopathological features and DNA analysis, the patient was diagnosed with pycnodysostosis accompanied with sporadic MTC.

## 3 Discussion

Pycnodysostosis is a rare autosomal-recessive disease causing osteosclerosis due to decreased bone resorption. It is characterized by reduced stature, osteosclerosis, acroosteolysis of the distal phalanges, frequent fractures, hypoplasia of the clavicle, skull deformities with delayed suture closure, as well as oral abnormalities including malposed teeth, proneness to dental caries, a highly arched bone palate, and a long soft palate with a long uvula. There may also be trunk deformities, for instance, kyphosis, lordosis, or a narrow chest. Occasionally, exophthalmos and blue sclera are present.^[3]^ Because short stature is an essential feature of the disease, many patients are usually referred to the pediatric unit with failure to thrive or to dentists because of oral deformities that severely affect their daily life. Thus, radiological techniques to confirm osteosclerosis and acroosteolysis combined with genetic analysis are required for diagnostic purposes. The patient presented in this report was diagnosed with typical pycnodysostosis as described in the literature; however, her case is the first exhibiting pycnodysostosis accompanied by MTC, which is also a rare neuroendocrine tumor.

MTC, which is further categorized into sporadic MTC and MEN2, originates from the parafollicular C cells and exhibits histopathologic and clinical characteristics that differ from papillary or follicular carcinomas of thyroid origin. A solitary thyroid nodule is the most common feature of MTC. Clinically, patients with MEN2 may also develop parathyroid hyperplasia, pheochromocytoma, parathyroid adenoma, mucosal neuroma, or pheochromocytoma. Moreover, most MEN2 cases derive from a variation in the *RET* proto-oncogene. In this reported case, possibilities of parathyroid hyperplasia or adenoma were excluded based on an ultrasound examination of the parathyroid and laboratory tests revealing normal serum calcium, phosphate, and parathyroid hormone levels. It was also less likely to be pheochromocytoma, based on the normal blood pressure and computed tomography examination of the adrenal gland, or mucosal neuroma, according to the physical examination. Further DNA sequencing of the *RET* proto-oncogene did not detect any mutation. Therefore, a sporadic MTC was diagnosed.

Although MTC accounts for only 5% to 10% of thyroid carcinomas, its prognosis is severer than more-well-differentiated thyroid carcinomas. Moreover, in most patients, the disease is always diagnosed accompanied with metastases to regional lymph nodes or even to the lungs, liver, and bone tissue.^[4]^ In this reported case, multiple metastases were also detected in the extrathyroidal extension and lymph nodes in the right-sided neck (7 out of 9 nodes). Consistently, 1 year after total thyroidectomy, lymph node metastases consequent to MTC was considered based on laboratory test on calcitonin and CEA as well as ultrasonography examination. So far, this patient has been referred to the Department of Oncology for further treatment.

Pycnodysostosis is caused by mutations in the *CTSK* gene located on chromosome 1q21 encoding a lysosomal cysteine protease, cathepsin K, which is widely expressed in bone, ovary, heart, placenta, lung, skeletal muscle, colon, and small intestine. As a member of the papain-cysteine protease family, it is responsible for degrading bone matrix proteins, type I/type II collagen, osteopontin, and osteonectin and for bone remodeling. Cathepsin K is synthesized as an inactive precursor protein that requires removal of its N-terminal pro-region for activation. To date, at least 35 different mutations have been reported in the *CTSK* locus leading to nonsense, missense, frameshift, and splicing mutations, as well as small deletions or insertions. The majority of the mutations are located in the mature active domain of cathepsin K protein.^[5,6]^ Our patient exhibited compound heterozygous mutations in the *CTSK* gene (c.158delA, P.Asn53Thr/c.C830T, P.Ala277Val). The C830T mutation is a missense mutation located in the mature region and causes loss of function, which has been reported previously. The C158delA is a novel mutation leading to a reading frameshift and a truncated form of only 54 amino acids at the N-terminus. Indeed, compound heterozygous mutations of the *CTSK* gene have been frequently reported in patients with pycnodysostosis diseases (as reviewed by Xue et al^[5]^), which accounts for approximately 24.73% of the included patients.

To date, an increasing number of patients acquire multiple primary carcinomas because of environmental modifications or genetic predispositions. Under this particular circumstance, the possible association between the pathogenesis of pycnodysostosis and MTC is unclear. Although it may only be an unfortunate coincidence, this case still leads us to further consider any potential association between these 2 rare diseases. Both in vivo and in vitro studies show that cathepsin K protein is predominantly found within the thyroid follicular lumen and involved in liberating thyroxine from thyroglobulin; however, research on cathepsin K in para-follicular cells is absent.^[2,7,8]^ Thus, it will be difficult to speculate whether *CTSK*-deficiency or loss of function is directly affecting in the pathogenesis of MTC. Indeed, apart from its well-confirmed involvement in bone disease, cathepsin K is also highly expressed in gastric cancer, squamous cell carcinoma, basal cell carcinoma, breast tumors, lung cancer, melanomas, prostate tumors, and renal tumors and appears to have a positive role in promoting tumor progression.^[9,10]^ Other cathepsins (including cathepsins B, D, L, etc) also have the potential to accelerate tumorigenesis by promoting extracellular matrix (ECM) degradation. These lines of evidence appear to be controversial to what we have seen in this case report.^[11]^ However, Friedrichs et al^[7]^ proposed a compensatory role of cathepsin L for cathepsin K deficiency, in which case the cathepsin L protein level is enhanced when cathepsin K is absent. Therefore, it will be of interest to further evaluate the expression pattern of cathepsins K and L in thyroid tissue. Furthermore, cathepsin K is closely related to osteoclast function (being primarily responsible for bone matrix degradation by osteoclasts) and acts as a potential regulator of apoptosis and senescence to control osteoclast numbers in vivo.^[12,13]^ Considering that bone destruction in skeletal metastases may result from osteoclast-induced bone resorption,^[14]^ a *CTSK* mutation, in this case, will potentially affect the extent of MTC skeletal metastases. In particular, based on the clinical observation and the phenotypes of *CTSK*-deficient mice, cathepsin K loss of function may also lead to changes in the immune and hematopoietic systems. For example, a low-grade anemia was observed in this patient. Thus, there may be a potential correlation between the immune or hematopoietic system and MTC progression. The consideration of these findings will encourage us to further investigate the structure and function of this mutated cathepsin K protein, as well as its potential involvement in the pathogenesis of MTC, either directly or indirectly.

Notably, mounting evidence has revealed that thyroid hormones have profound effects on bone development, linear growth, and adult bone maintenance, including the inhibition of osteoclast formation and function.^[15]^ Cathepsin K, intensively expressed in osteoclasts, is essential for normal bone resorption. Recently, a large multinational, randomized, double-blind phase III study of odanacatib (a cathepsin K inhibitor) in postmenopausal women with osteoporosis was completed.^[16]^ Both thyroid hormone and cathepsin K were closely related to bone maintenance and disease, for example, osteoporosis. Certain findings have indicated the direct correlation of thyroid hormones and cathepsin K but are limited and controversial. Mikosch et al^[17]^ reported high cathepsin K levels detected in patients on suppressive L-thyroxine therapy. By contrast, Zhang et al^[18]^ have shown that TSH inhibits cathepsin K expression and osteoclastogenesis in RAW264.7 cells. Nerveless, in the present case, the *CTSK* gene was mutated and presumably underwent loss of function. Additionally, the serum TSH, free triiodothyronine, and free thyroxine levels were all in the normal range, which indicated that thyroid hormone likely did not affect the pathogenesis of pycnodysostosis. In addition, calcitonin was intensively secreted by MTC in this case. Calcitonin may protect against skeletal calcium loss by directly inhibiting bone resorption or indirectly inhibiting prolactin released from the pituitary gland. Therefore, theoretically, increased calcitonin and a mutated cathepsin K possibly have a synergistic effect on bone resorption.

In sporadic MTC cases, total thyroidectomy and central lymph node dissection should be performed. Lateral lymph node dissection is further required when invasion is identified. Few chemotherapeutic options are available for patients with metastatic MTC that cannot be cured by surgery.^[19]^ To date, no specific treatments have been developed for pycnodysostosis. It is critical to emphasize and promote supportive management and prophylactic measures to prevent fracture occurrence and maintain oral hygiene.^[20]^

## 4 Conclusions

In conclusion, the present study is the first to report a rare case of the coexistence of pycnodysostosis with novel *CTSK* gene mutation and sporadic MTC. Radiological techniques and genetic analysis played key roles in the definitive diagnosis.

## Supplementary Material

Supplemental Digital Content
