# Supplementary figures and images for: Pycnodysostosis with novel gene mutation and sporadic medullary thyroid carcinoma: A case report
Source: Medicine (Baltimore). 2017 Dec 15;96(50):e8730. doi: 10.1097/MD.0000000000008730 (PMC5815678; doi:10.1097/MD.0000000000008730)

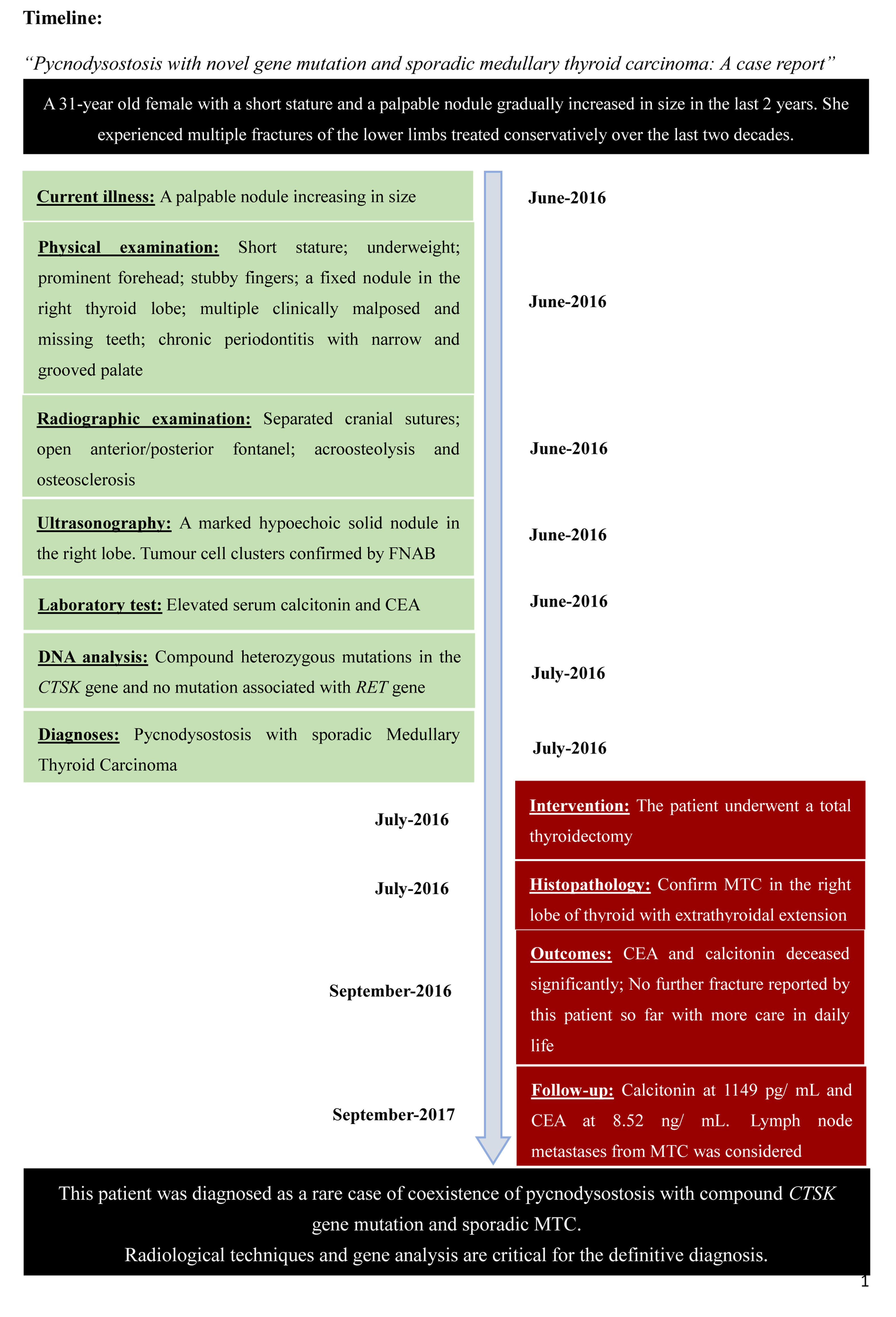

Supplement: Supplemental Digital Content [file medi-96-e8730-s001.tif]
